# Senescence‐based colorectal cancer subtyping reveals distinct molecular characteristics and therapeutic strategies

**DOI:** 10.1002/mco2.333

**Published:** 2023-07-26

**Authors:** Min‐Yi Lv, Du Cai, Cheng‐Hang Li, Junguo Chen, Guanman Li, Chuling Hu, Baowen Gai, Jiaxin Lei, Ping Lan, Xiaojian Wu, Xiaosheng He, Feng Gao

**Affiliations:** ^1^ Department of Genaral Surgery （Colorectal Surgery） The Sixth Affiliated Hospital Sun Yat‐sen University Guangzhou China; ^2^ Guangdong Provincial Key Laboratory of Colorectal and Pelvic Floor Disease The Sixth Affiliated Hospital, Sun Yat‐sen University Guangzhou China; ^3^ Biomedical Innovation Center The Sixth Affiliated Hospital,Sun Yat‐sen University Guangzhou China

**Keywords:** cancer subtype, colorectal cancer, multi‐omics, senescence, therapeutic strategy

## Abstract

Cellular senescence has been listed as a hallmark of cancer, but its role in colorectal cancer (CRC) remains unclear. We comprehensively evaluated the transcriptome, genome, digital pathology, and clinical data from multiple datasets of CRC patients and proposed a novel senescence subtype for CRC. Multi‐omics data was used to analyze the biological features, tumor microenvironment, and mutation landscape of senescence subtypes, as well as drug sensitivity and immunotherapy response. The senescence score was constructed to better quantify senescence in each patient for clinical use. Unsupervised learning revealed three transcriptome‐based senescence subtypes. Cluster 1, characterized by low senescence and activated proliferative pathways, was sensitive to chemotherapeutic drugs. Cluster 2, characterized by intermediate senescence and high immune infiltration, exhibited significant immunotherapeutic advantages. Cluster 3, characterized by high senescence, high immune, and stroma infiltration, had a worse prognosis and maybe benefit from targeted therapy. We further constructed a senescence scoring system based on seven senescent genes through machine learning. Lower senescence scores were highly predictive of longer disease‐free survival, and patients with low senescence scores may benefit from immunotherapy. We proposed the senescence subtypes of CRC and our findings provide potential treatment interventions for each CRC senescence subtype to promote precision treatment.

## Graphical abstract

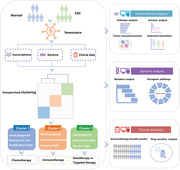

## 1 INTRODUCTION

Colorectal cancer (CRC), the most common digestive malignancy with high mortality, is a noticeable public health issue in both developed and developing countries.^1^, ^2^, ^3^ Nowadays, despite a better understanding of CRC mechanisms and great advances in treatment options,^4^, ^5^ the 5‐year overall survival (OS) of CRC remains less than 65%.^3^ So far, approximately 40% of patients with CRC still fail to achieve a more significant outcome benefit from routine therapeutic strategy including surgery combined with chemotherapy and radiotherapy.^6^, ^7^

One of the major challenges in CRC treatment is tumor heterogeneity, which refers to the presence of diverse subpopulations of cancer cells with different molecular and genetic profiles within a single tumor. In recent years, researchers have found that tumor heterogeneity can promote the evolution and adaptation of tumors and hinder the effectiveness of individualized drug strategies,^8^, ^9^, ^10^, ^11^ thus becoming a major obstacle to cancer treatment. Therefore, it is necessary to comprehensively dissect the tumor heterogeneity of CRC and develop effective molecular biomarkers for individualized treatment. Recent advances in high‐throughput sequencing and multi‐omics analysis have enabled the identification of molecular subtypes of CRC with distinct clinicopathological features and treatment outcomes.^12^ However, the biological basis and clinical implications of CRC heterogeneity remain incompletely understood, and there is a critical need for further exploration and validation of molecular biomarkers for precision medicine in CRC.

It is known that cancer cells are characterized by a series of hallmarks different from non‐neoplastic cells.^13^ With the deepening understanding of cancer mechanisms, a list of cancer hallmarks has also been refined as Hanahan edited and expanded in 2022.^14^ Among them, a new cancer hallmark of senescent cells^14^ has attracted our attention in particular. Cellular senescence used to be considered a protective mechanism to maintain tissue homeostasis.^15^, ^16^, ^17^, ^18^ However, more and more evidence suggested that in certain contexts, senescent cells could promote the occurrence and development of tumors in various ways.^14^, ^15^, ^16^, ^17^, ^18^, ^19^ Besides, senescence may affect the fate of cancer cells as well as other types of cells in the tumor microenvironment (TME), thereby modulating cancer hallmarks and tumor phenotypes.^16^, ^20^, ^21^, ^22^ Sun et al. found that senescence was a marker of gastric cancer that affected the prognosis and therapeutic effect of patients.^23^ Lung adenocarcinoma senescence subtypes identified by Shukla et al suggested that higher senescence cell lines were more resistant to drugs.^24^ Wang et al. established aging subtype and young subtype of CRC,^25^ however, this study utilized only publicly available datasets for the analysis and the training set included only colon cancer data, indicating that the findings may not be fully representative CRC. Our knowledge about the senescent characteristics of CRC is still preliminary, and the functions and clinical effects of senescence at different levels are largely unknown. Furthermore, it is unclear whether the senescent hallmarks of CRC patients can be used as prognostic biomarkers to guide clinical treatment decisions.

To address these critical problems, we comprehensively analyzed the senescent heterogeneity in CRC through a large in‐house RNA‐seq cohort (*n* = 587) and multiple public datasets. We identified three distinct senescence subtypes with significantly different biological characteristics, prognosis, and treatment response. We further constructed a senescent scoring system and assessed whether senescent scores can be used as prognostic biomarkers to guide clinical treatment decisions. Our research proposed CRC senescence subtypes which have good applicability in the Chinese population, as well as universality and reliability in other populations. Overall, our study contributed to the role of senescence in CRC and provided a foundation for the development of precision medicine strategies for CRC patients.

## 2 RESULTS

### 2.1 Identification of senescence as a hallmark of CRC

**FIGURE 1:**
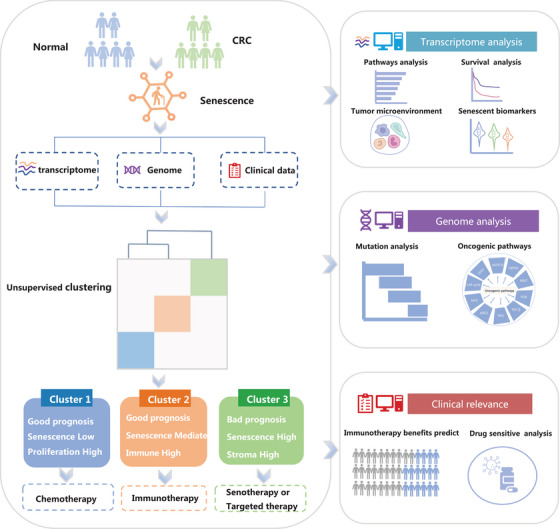
Schema flow chart of the study. The analysis included multi‐omics data from transcriptomic, genomic, and whole slide images of more than 2500 colorectal cancer (CRC) patients. Senescence subtypes were established by unsupervised clustering, and multi‐omics data were utilized to further analyze the biological characteristics, tumor microenvironment, mutational landscape, and drug sensitivity of the senescence subtypes. Finally, the senescence scoring system senescore was developed and its clinical prognosis and therapeutic efficacy were validated in multiple CRC cohorts.

The workflow of our study is shown in Figure 1. First, we obtained a complete list of senescence‐related pathways from MSigDB (Version 7.5.1, https://www.gsea‐msigdb.org/gsea/msigdb) using the keywords “senescence”, “senescent” and “aging” (Table S1). To illustrate the role of senescence in CRC, we first compared the transcriptome data of the CRC sample with its matched normal tissue from the ICGC‐ARGO cohort. We could see that the senescence pathways in CRC were more abundant than in normal tissues through the heatmap (Figure S1A). The UMAP plot showed that the senescent pathways could also distinguish the CRC and normal tissue (Figure S1B). These results indicate that senescence is a major characteristic of CRC.

To investigate the core senescence genes of CRC, we screened a total of 3,859 differentially expressed genes (DEGs) between CRC and paired adjacent normal tissue. We also conducted univariate Cox analysis on all CRC genes from the ICGC‐ARGO dataset and obtained 12,000 survival genes related to disease‐free survival (DFS). In addition, we obtained 1,409 senescence genes from Peters et al.’s study.^26^ Finally, we intersected DEGs, survival genes, and senescence genes and finally established the 77 core senescence genes (CSGs) (Figure S1C,D).

### 2.2 Identification of senescence subtypes based on the CSGs

**FIGURE 2:**
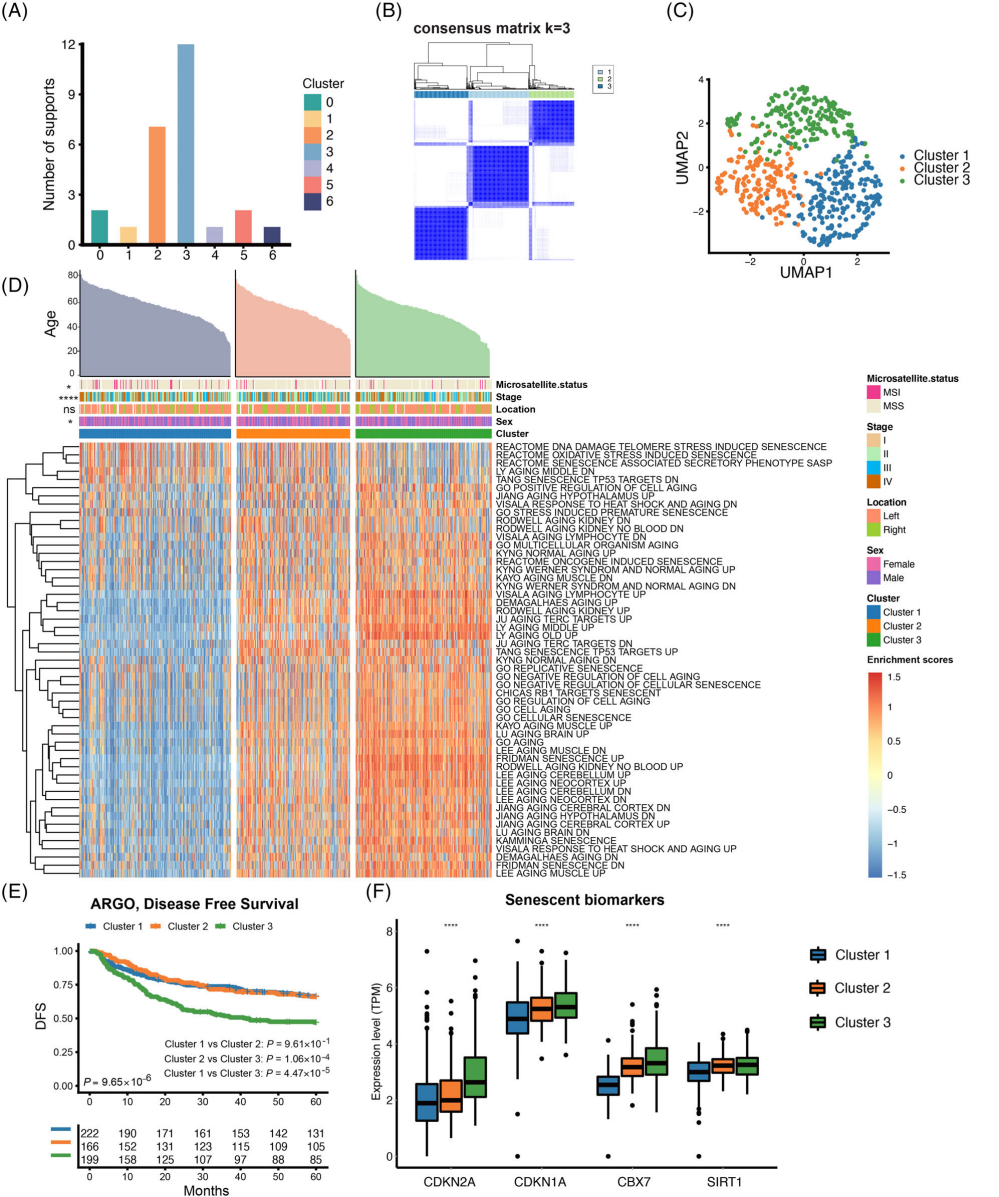
Identification of the senescence subtypes in colorectal cancer (CRC). (A) Nbclust was used to determine the optimal number of clusters. (B) Unsupervised clustering of patients in the ICGC‐ARGO cohort based on 77 CSGs categorized patients into three senescence subtypes. Labels 1, 2, and 3 refer to the three different categories that patients can be classified into cluster 1, cluster 2, and cluster 3, respectively. (C) UMAP analysis revealed that the expression of 77 CSGs represented distinct senescence subtypes. (D) Heatmap showed the enrichment of senescence‐related pathways in three senescence subtypes. Age, sex, tumor location, TNM stage, and microsatellite status were shown above the heatmap. The asterisks represented the statistical *p‐*value (ns, not significant; *p < 0.05; **p < 0.01; ***p < 0.001; **** *p* < 0.0001). (E) Kaplan–Meier curves showed a significant disease‐free survival difference among the three clusters. (F) Senescent biomarkers expressed differently among three senescence subtypes in the ICGC‐ARGO cohort.

To reveal the underlying molecular features of the CRC senescence subtypes, we performed an unsupervised clustering analysis based on CSGs expression in the ICGC‐ARGO dataset. With the “Nbclust” R package, we determined that k = 3 was the best cluster number, so we divided the patients with CRC into three senescence subtypes, which were named cluster 1, cluster 2, and cluster 3, respectively (Figure 2A,B). We utilized the deep learning‐based cancer subtype classification (DeepCC) algorithm^27^ to make predictions of the senescence subtypes in other cohorts. UMAP analysis showed that there were significant differences in transcriptome profiles of 77 CSGs among the three senescence subtypes (Figure 2C). Then, we compared the activation of senescent pathways on three senescence subtypes. We found cluster 3 displayed significantly higher activation in senescent pathways while cluster 1 showed lower activation. (Figure 2D and Figure S2A). As for 50 cancer hallmark pathways, we interestingly found that cluser1 was highly enriched in the G2M checkpoint, *MYC* and other cell cycle‐related pathways. Cluster 2 displayed significantly higher activation in interferon, inflammation response and other immune‐related pathways. Cluster 3 was highly enriched in tumor development and signaling pathways such as the epithelial‐mesenchymal transition (EMT), NOTCH and *PI3K* (Figure S3A,B). In addition, survival analysis showed that DFS curves were significantly differentiated among the three senescence subtypes (*p* < 0.0001), and cluster 3 presented the worst prognosis (Figure 2E and Figure S2D,E). This result was validated in the TCGA‐CRC cohort (Figure S2B). Next, we reviewed the published high‐quality senescence‐related articles in recent 5 years and obtained the commonly used senescent markers. We found that senescent markers such as *CDKN1A*,^28^, ^29^ *CDKN2A*,^29^, ^30^ *CBX7* ^30^, ^31^ and *SIRT1* ^30^ were highly expressed in cluster 2 and cluster 3, and this result was consistent in the TCGA‐CRC cohort (Figure 2F and Figure S2C).Overall, we identified three distinct senescence subtypes with varying activation levels of senescent and cancer hallmark pathways, as well as prognosis based on survival analysis.

### 2.3 The landscape of genetic variation of senescence subtypes in CRC

**FIGURE 3:**
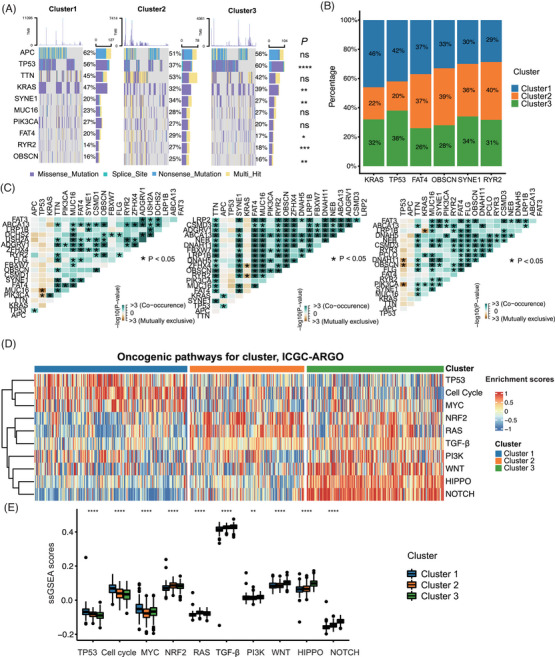
Expression variation of three senescence subtypes. (A) The waterfall plot showed the top 10 gene mutations with the highest frequency of mutations among the three senescence subtypes (TCGA‐CRC cohort). (B) The stacked histogram showed significant proportions of gene mutation rates among the three senescence subtypes (TCGA‐CRC cohort). (C) Mutation characteristics of three senescence subtypes in the TCGA‐CRC cohort. (D) Heatmap showed the enrichment of ten important oncogenic pathways among the three senescence subtypes (ICGC‐ARGO cohort). (E) Boxplot showed score variations in ten important oncogenic pathways among the three senescence subtypes (ICGC‐ARGO cohort).

We showed the top 10 mutated genes of three senescence subtypes and their mutation rates. Among them, the mutation rates of *TP53*, *KRAS*, *SYNE1*, *FAT4*, *RYR2*, and *OBSCN* were statistically different in the three clusters (Figure 3A,B). Specifically, the mutation rate of *KRAS* was higher in cluster 1, while *FAT4*, *OBSCN*, *SYNE1*, and *RYR2* mutation rate was higher in cluster 2, and the mutation rate of *TP53* was higher in cluster 3 (Figure 3B). Co‐occurrence and mutual exclusivity of the most common mutations were calculated to indicate the biological cooperativity of some mutation events. There were significant co‐mutations between *PIK3CA* and *KRAS*, *SYNE1* and *TTN*, and *FAT4* and *MUC16* in cluster 1 (*p* < 0.05). In cluster 2, there were significant co‐mutations between *OBSCN* and *TTN*, *RYR2* and *FAT4*, and *ZFHX4* and *MUC16* (*p* < 0.05). In cluster 3, there were significant co‐mutations between *OBSCN* and *MUC16*, and *FAT3* and *SYNE1* (*p* < 0.05) (Figure 3C). Furthermore, we utilized the ssGSEA algorithm to calculate scores for 10 oncogenic pathways,^32^ and examined their distribution across three senescence subtypes. Interestingly, we could see that cluster 1 was highly enriched in *TP53*, *MYC* pathways and other cell cycle‐related pathways. Cluster 2 displayed significantly higher activation in *NRF2* and *RAS* pathways, while cluster 3 was highly enriched in *PI3K*, HIPPO, WNT and NOTCH pathways (Figure 3D,E). A similar result was verified in the TCGA‐CRC cohort (Figure S4A,B).

### 2.4 Immune profiling of senescence subtypes

**FIGURE 4:**
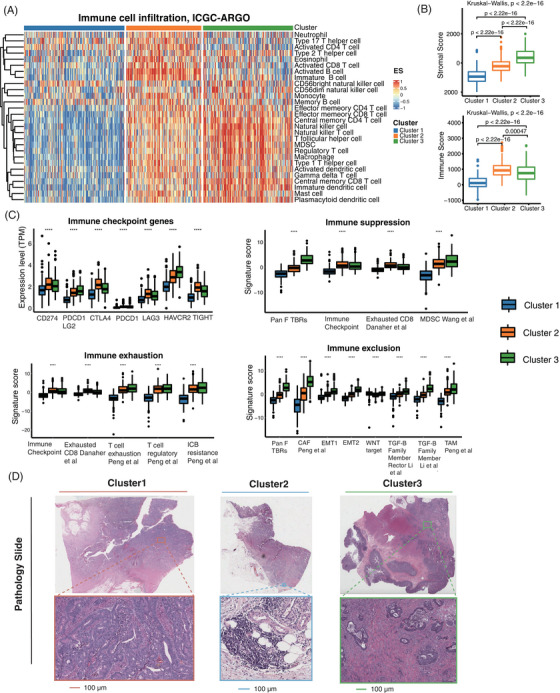
Variation of tumor immune microenvironment in three senescence subtypes. (A) Heatmap showed the infiltration abundance of immune cells evaluated by ssGSEA for three senescence subtypes (ICGC‐ARGO cohort). (B) Boxplot showed stromal score and immune score among the three senescence subtypes (ICGC‐ARGO cohort). (C) Boxplot showed the differences in immune checkpoint genes, immune suppression, immune exclusion, and immune exhaustion among the three senescence subtypes (ICGC‐ARGO cohort). (D) Representative pictures of pathological HE staining of three senescence subtypes (TCGA Pathology Slide).

Based on the above findings, we determined that there were significant biological differences between the three senescence subtypes. We further investigated the tumor immune microenvironment of the three senescence subtypes. We utilized the ssGSEA to analyze the immune cell infiltration of three senescence subtypes, in which the overall infiltration abundance of cluster 2 and cluster 3 was significantly higher than that of cluster 1 (Figure 4A). We also found that cluster 2 had the highest immune score (*p* < 0.0001) and cluster 3 had the highest stromal score (*p* < 0.0001) by the ESTIMATE algorithm (Figure 4B). In addition, to reveal the tumor‐immune interactions and explore the potential of immunotherapy for senescence subtypes, we analyzed the expression of immune checkpoint genes and published signatures involved in immune suppression, immune exclusion and immune exhaustion.^33^ The gene expressions of *CD274*(PD‐L1), *PDCD1*(PD‐1), *CTLA4*, and *LAG3* were higher in cluster 2, while myeloid‐derived suppressor cells (MDSC), cancer‐associated fibroblasts (CAFs), EMT, T cell exhaustion and other immune suppression/exclusion/exhaustion related signatures were most expressed in cluster 3 (Figure 4C). Based on the nine‐tissue classification result, different subtypes of clusters showed significant variable composition in their whole slide image (WSI). The violin plot demonstrated that cluster 1 patients had a higher proportion of colorectal adenocarcinoma epithelium (TUM) in the pathological images, while cluster 2 and cluster 3 patients had higher lymphocytes (LYM) percentage and higher cancer‐associated stroma (STR) percentage, respectively (Figure S5). Three representative WSIs for each cluster were displayed (Figure 4D). It can be observed that in Cluster 1 patients, the tumor cells proportion was higher in the tumor pathological sections, while in Cluster 2 patients, the WSI had more immune cell components, such as lymphocytes. Cluster 3 patients had more cancer‐associated stroma enrichment. In summary, we identified significant biological differences among the three senescence subtypes and their respective tumor immune microenvironments. Also, we used the DeepCC algorithm^27^ to predict CMS subtypes. The relationship between the senescence subtypes CMS was analyzed, among which cluster 1 accounted for higher proportion of CMS2 and CMS3, cluster 2 accounted for a relatively high proportion in CMS1, and cluster 3 was overrepresented in the CMS4 subtype (Figure S4C,D).

### 2.5 Predictive value of senescence subtypes for immunotherapy and drug sensitivity analysis

**FIGURE 5:**
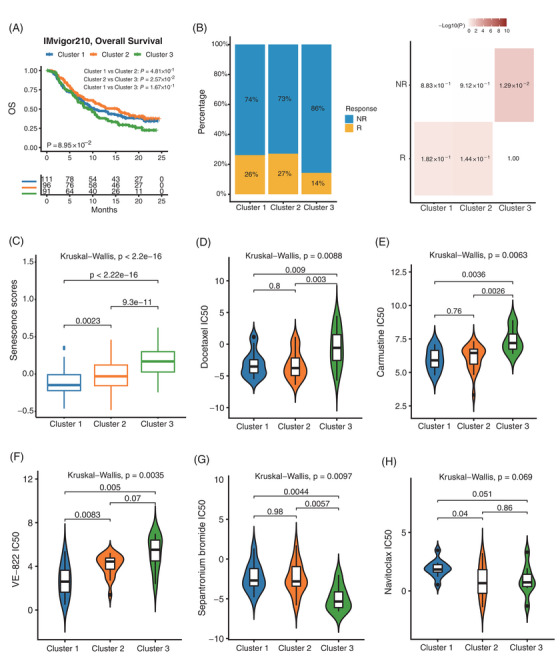
Validation of the therapeutic benefits of the three senescence subtypes. (A) The Kaplan‐Meier curve showed the overall survival of three senescence subtypes in the IMvigor210 database. (B) The stacked histogram showed the proportion of immunotherapy responses of three senescence subtypes in the IMvigor210 database. (C) The correlation between senescence subtypes and senescence scores in the IMvigor210 database. (D–H) IC50 values of three senescence subtypes on Docetaxel (D), Carmustine (E), VE‐822 (F), Sepantronium bromide (G), and Navitoclax (H).

We then used the IMvigor210 dataset to explore the relationship between senescence subtypes and immunotherapy. Survival analysis showed that cluster 1 and cluster 2 had a significantly higher proportion of responders in immunotherapy with a better prognosis (Figure 5A–C). To further investigate the clinical application of senescence subtypes, the Genomics of Drug Sensitivity in Cancer (GDSC) database was used to analyze the differences in drug sensitivity among three senescence subtypes. We observed that the Docetaxel and Carmustine chemotherapeutic agents were more resistant in cluster 3 than the other two senescence subtypes (Figure 5D,E). Besides, we found that the drug VE‐822 was effective in treating cluster 1 compared to other subtypes, which could attenuate the ATR signaling pathway^34^, ^35^, ^36^ (Figure 5F). We also found that of all the drugs in the GDSC dataset, only one drug called Sepantronium bromide was most sensitive to cluster 3, which can target DNA topoisomerase^37^, ^38^ (Figure 5G). Interestingly, as an anti‐aging drug, Navitoclax^15^, ^30^, ^39^ was more sensitive to cluster 2 and cluster 3 (Figure 5H). The other statistically significant drugs in the three senescence subtypes were shown in Figure S6 and Table S2. These findings could have important clinical implications for tailoring therapeutic approaches to the different senescence subtypes of CRC.

### 2.6 Construction of a senescence scoring model

To accurately quantify the senescent characteristics of individual patients, we constructed a senescence scoring model to quantify the level of senescence in individual CRC patients. The ICGC‐ARGO (*n* = 587) was used as the training cohort. 20 top expressed genes were screened out from 77 CSGs to further construct the LASSO model. Finally, 7 CSGs were selected and weighted sum for the construction of senescence scoring model (Figure S7A). The risk score was imputed as follows: (0.0178 × *IGFBP3*) + (0.0288 × *IGFBP7*) + (0.0168 × *BHLHE40*) + (0.106 × *TTYH3*) + (0.0077 × *TIMP1*) + (0.119 × *TAGLN*) + (0.0277 × *PLOD3*).

### 2.7 Evaluation of the senescence scoring model

**FIGURE 6:**
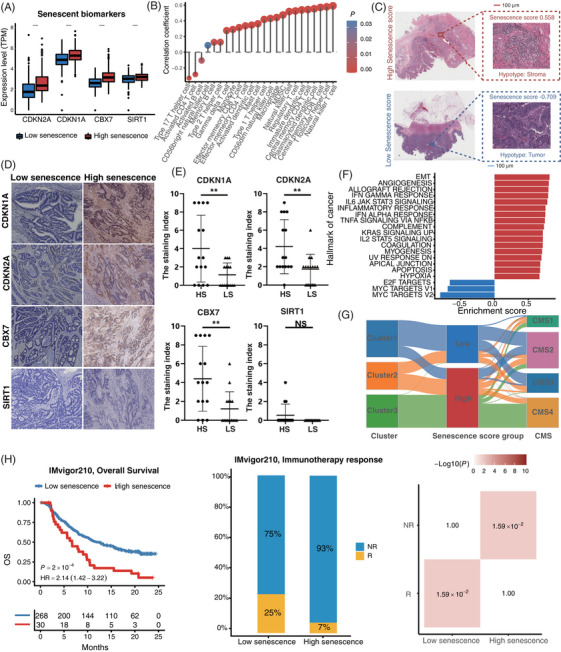
Validation of the therapeutic benefits of the senescence scoring system. (A) Difference of senescent biomarkers between low and high senescence score groups. (B) The dot plot showed the correlation between the senescence score and the infiltration abundance of immune cells evaluated by ssGSEA (ICGC‐ARGO cohort). (C) Image representing the pathological HE staining variation between the high and low senescence score groups (TCGA Pathology Slide). (D) Immunostaining analyses of senescence biomarkers between high and low senescence groups. Fifteen low senescence group and 15 high senescence group cases were collected from the ICGC‐ARGO cohort. The image was captured at 10x magnification. (E) Boxplots illustrated the IHC scores in two different groups based on IHC analysis. HS: high senescence group; LS: low senescence group. The asterisks represented the statistical *P* value (ns, not significant; *p < 0.05; **p < 0.01; ***p < 0.001; ****p < 0.0001) (F) Bar plot showed hallmark difference between low and high senescence score groups (ICGC‐ARGO cohort). (G) Sankey plot showed the correlation among senescence subtypes, senescence score groups and consensus molecular subtypes (CMS) (ICGA‐ARGO cohort). (H) The Kaplan‐Meier curve showed the overall survival of low and high senescence score groups in the IMvigor210 database. The stacked histogram showed the proportion of immunotherapy responses of low and high senescence score groups in the IMvigor210 database.

Survival analysis showed that patients with high senescence scores were associated with worse outcomes (hazard ratio [HR] = 2.65, 95% confidence interval [95%CI] = 1.94–3.61, *p* < 0.001) in the ICGC‐ARGO cohort (Figure S7B and Table S3). To confirm the robustness of the senescence score across different cohorts, we applied our model in other datasets and the results confirmed that patients with high senescence score had significantly shorter DFS (TCGA: HR = 1.79, 95%CI = 1.34–2.39, *p* < 0.001; GSE39582: HR = 1.61, 95%CI = 1.22–2.11, *p* < 0.001; Meta‐GEO: HR = 1.83, 95%CI = 1.27–2.64, *p* = 0.001) (Figure S7C–E). In addition, the senescence score was identified as an independent and powerful prognostic factor when evaluated in multivariate Cox regression models (Figure S7F and Table S3). For better clinical application, we combined senescence score with age and TNM stage to generate a nomogram (Figure S7G). The calibration curve showed that there was good concordance between the predicted and observed values of 3‐year and 5‐year DFS in ICGC‐ARGO cohorts​, reflecting the accurate prediction ability (Figure S7H). We demonstrated that the nomogram had a better prognostic ability than TNM by the area under the curve (AUC) of the time‐dependent receiver operating characteristic curve (ROC) (Nomogram: AUC = 0.84; TNM stage: AUC = 0.80) (Figure S7I). In addition, the expression of senescent markers *CDKN1A*, *CDKN2A*, *CBX7* and *SIRT1* was higher in patients with high senescence score (Figure 6A and Figure S8A). Besides, high senescence scores seemed to be associated with high immune infiltrating cells (Figure 6B and Figure S8G). The histopathological section confirmed that the high senescence scores group showed higher infiltration of immune cells and stromal cells (Figure 6C). Immunohistochemical analysis of tissue samples from our ICGC‐ARGO cohort, consisting of 30 CRC patients, demonstrated detectable expression of *CDKN1A*, *CDKN2A*, *CBX7*, and *SIRT1* using specific antibodies (Figure 6D,E). Notably, the high senescence group exhibited a significantly elevated positive expression rate of *CDKN1A*, *CDKN2A*, and *CBX7* compared to the low senescence group. However, no significant difference in *SIRT1* expression was observed between the two groups. The protein expression levels of these senescence biomarkers were relatively consistent with their corresponding gene expression levels (Figure 6A). Hallmarks of cancer pathways with statistical significance in high and low senescence groups are shown in Figure 6F. We observed that EMT, angiogenesis, KRAS, and hypoxia‐related pathways were highly enriched in the high senescence group, while the low senescence group was mainly enriched in proliferation‐related pathways such as MYC and G2M checkpoint. These findings suggest that senescence may contribute to different molecular mechanisms underlying cancer development and progression.We then used the Sankey plot to describe the association among senescence subtypes, CMS subtypes and senescence scores (Figure 6G and Figure S8B). The senescence score of cluster 3 was significantly higher than that of cluster 1 and cluster 2, and CMS 4 was significantly associated with a higher senescence score (Figure S8C–F). Besides, we explored whether our senescence score model could predict the treatment efficiency of immunotherapy, and we divided patients into high and low senescence score groups in the IMvigor210 dataset and assessed differences in their OS. We found that immunotherapy significantly improved the OS rate in patients with low senescence scores (*p* = 0.0002), and a greater proportion of patients in the low group responded to immunotherapy (*p* = 0.0159) (Figure 6H). These results suggested that senescence score can be used as a potential biomarker to help clinicians and select the appropriate patient for immunotherapy.

## 3 DISCUSSION

More and more studies have shown that the accumulation of senescent cells in tissues and organs with aging and at sites of various pathologies was largely detrimental.^21^ In elderly patients with CRC, the senescence phenotype induced immune activation and proinflammatory states that negatively affect disease outcomes.^40^ This is the first comprehensive analysis of heterogeneity of senescence in CRC with a large sample and multi‐cohort validation. We confirmed senescence as an important characteristic of CRC. The differences in senescent‐related pathway enrichment between cancer‐adjacent and CRC tissues may have significant implications for our understanding of cancer development and progression. It is important to note that in our study, each patient's tumor tissue and adjacent tissue were paired, ensuring consistent tissue age. Nonetheless, our analysis revealed significant differences in the enrichment of senescent‐related pathways between the two tissue types, despite their similar ages. These findings suggest that CRC occurrence may not be influenced by age‐related physiological changes, challenging the traditional belief that CRC primarily affects the elderly and emphasizing the need for broader prevention and treatment measures. Our study provides insight that CRC can occur at any age, highlighting the importance of regular screening.

In addition, we identified three senescence subtypes in patients with CRC. The three senescence subtypes differed significantly in biological features, TME, and mutation landscape. Cluster1 with low senescence was highly enriched in the cell cycle, *MYC*, and other pathways. We, therefore, defined it as a proliferative/senescence low subtype. By utilizing the GDSC database, we found that cluster 1 was more sensitive to the VE‐822, which reduced tumor cell survival by attenuating the ATR signaling pathway.^41^ We knew that cisplatin triggered a DNA damage response by activating the ATR‐CHk1 pathway, which in turn helped to induce cisplatin resistance.^34^, ^42^ The use of VE‐822 may inhibit ATR‐Chk1 signaling and thus achieve reversal of cisplatin resistance in some cancers.^34^ We speculate that the combination of conventional chemotherapeutic agents with VE‐822 may achieve more effective therapeutic results for cluster 1. Cluster 2 was highly enriched for immune‐related pathways and expressed a higher level of *CD274* (PD‐L1), *PDCD1* (PD‐1), *CTLA4*, and *LAG3*. So we defined cluster 2 as an immunosensitive/senescence intermediate subtype, and the validation in the IMvigor210 dataset further confirmed the better benefit of immunotherapy for PD‐L1 in cluster 2 CRC patients. Cluster 3 was characterized by the highest senescence, higher stromal infiltration, and poor survival prognosis. Cluster 3 was highly enriched in tumor development pathways such as EMT, WNT, HIPPO, and molecules such as MDSC, CAFs, and T cell exhaustion were most expressed in cluster 3. We defined cluster 3 as a stromal/senescence high subtype, for which conventional chemotherapeutic agents and immunotherapy had low therapeutic effects on it. In the GDSC database, cluster 3 only had a significant therapeutic effect on the drug Sepantronium‐bromide (YM‐155), which was an effective inhibitor of survivin‐targeting DNA topoisomerases.^37^, ^38^ Navitoclax is an anti‐aging agent, and it has been reported that Navitoclax can reverse senescence to some extent,^15^, ^30^, ^39^ with clusters 2 and 3 being sensitive to it. We suspected that cluster 3 was more suitable for some specific targeted therapy, especially senotherapy.

The CMS classification system provides a more accurate way of classifying CRC based on gene expression profiles.^12^ However, it cannot fully reflect the complexity and heterogeneity of CRC. Senescence subtypes have distinct advantages over CMS subtypes in improving cancer classification and guiding treatment decisions. Senescence subtypes can provide personalized treatment options and more accurate prediction of tumor prognosis. Targeted therapies tailored to each senescence subtype can result in more effective treatments with fewer side effects, thus improving the quality of life for cancer patients. Understanding senescence subtypes is crucial in achieving more precise diagnosis and treatment, ultimately improving the clinical management of cancer.

In addition, we developed a senescence score model consisting of seven CSGs to assess the level of senescence in CRC, which was easier to translate into clinical practice. Our comprehensive analysis showed that the senescence score had an important clinical correlation and it was a robust prognostic biomarker. More importantly, the senescence score was proven to be an independent factor for DFS. In the IHC results, we found that *CDKN1A*, *CDKN2A*, and *CBX7* protein expression was significantly higher in the high senescence group compared to the low senescence group. However, there was no statistically significant difference in *SIRT1* protein expression between the two groups, possibly due to its relatively low expression level and the influence of post‐transcriptional modifications, post‐translational modifications, and metabolic homeostasis. Besides, we observed that a high senescence score correlated with a high stromal score, high EMT, and high content of CAFs. Also, some studies found that high stromal infiltration was closely associated with senescence.^43^, ^44^, ^45^ Stromal cells like CAFs, can alter the TME by secreting cytokines and other bioactive molecules, which can induce cellular senescence and exacerbate the instability of the TME.^46^, ^47^ Currently, targeted drugs against CAFs and high stroma of tumors are under development, but none have been approved for tumor treatment yet.^48^ Nevertheless, with a deeper understanding of the TME, targeted therapy against CAFs may become an important strategy for treating tumors.

Moreover, CRC patients with low senescence scores were more likely to benefit from immunotherapy. Therefore, the senescence score model was also a candidate biomarker to evaluate the therapeutic benefit of PD‐L1, and patients with high senescence scores may not be suitable for immunotherapy due to potential drug resistance and immune‐related adverse event.

Our research had some limitations. First, this is a retrospective study, although we validated the senescence subtypes and senescence scores in multiple independent datasets. Future validation of senescence subtypes and senescence scoring system should be performed in a prospective multicenter cohort. In addition, clinical evaluation of drug sensitivity tests is needed. We look forward to conducting clinical trials and transforming our results into clinical practice.

In conclusion, we proposed senescence subtypes for CRC patients. Our analysis showed that the three senescence subtypes had their prominent characteristics and were appropriate for different therapeutic strategies. The proposed senescence subtypes provide a new basis for precise diagnosis and treatment of CRC patients, paving the way for developing more rational treatment recommendations and facilitating personalized cancer therapy.

## 4 MATERIALS AND METHODS

### 4.1 Data acquisition and processing

The workflow of this study was shown in Figure 1. In total, seven eligible CRC cohorts with high‐quality gene expression and clinical data were gathered in this study for analysis. Our in‐house ICGC‐ARGO cohort included the RNA‐seq data from 587 CRC tissue and paired adjacent normal tissue, which was collected from the Sixth Affiliated Hospital of Sun Yat‐sen University (https://www.icgc‐argo.org/page/89/project‐list). The ICGC‐ARGO cohort was selected as the discovery cohort, while TCGA‐CRC, GSE17538, GSE14333, GSE33113, GSE37892, and GSE39582 were used for validation (Table 1). Public gene‐expression data and full clinical annotation were obtained from the Gene‐Expression Omnibus (GEO, https://www.ncbi.nlm.nih.gov/geo/) and the Cancer Genome Atlas (TCGA, https://portal.gdc.cancer.gov/).

**TABLE 1:** Characteristics of cohorts included in this study.

Characteristics	Training cohort ICGC‐ARGO	Validation‐1 TCGA‐CRC	Validation‐2 GSE39582	Validation‐3 Meta‐GEO
Number of patients	587	618	566	742
Mean age	58.25	66.27	66.85	66.47
Gender				
Male	342 (58.26%)	289 (46.76%)	310 (54.77%)	397 (53.50%)
Female	245 (41.74%)	329 (53.24%)	256 (45.23%)	345 (46.50%)
TNM stage				
Stage I	66 (11.24%)	105 (16.99%)	33 (5.83%)	72 (9.70%)
Stage II	201 (34.24%)	227 (36.73%)	264 (46.64%)	329 (44.34%)
Stage III	152 (25.89%)	179 (28.96%)	205 (36.22%)	224 (30.19%)
Stage IV	160 (27.26%)	88 (14.24%)	60 (10.60%)	117 (15.77%)
NA	8 (1.36%)	19 (3.07%)	4 (0.71%)	0
Tumor location				
Left	412 (70.19%)	349 (56.47%)	342 (60.42%)	233 (31.40%)
Right	136 (23.17%)	269 (43.53%)	224 (39.58%)	185 (24.93%)
NA	39 (6.64%)	0	0	324 (43.67%)
DFS event				
Yes	245 (41.74%)	196 (31.72%)	248 (43.82%)	160 (21.56%)
No	342 (58.26%)	422 (68.28%)	314 (55.48%)	485 (65.36%)
NA	0	0	4 (0.71%)	97 (13.08%)
Microsatellite status				
MSI	53 (9.03%)	188 (30.42%)	75 (13.25%)	34 (4.58%)
MSS	496 (84.50%)	427 (69.09%)	444 (78.45%)	107 (14.42%)
NA	38 (6.47%)	3 (0.49%)	47 (8.30%)	601 (80.99%)

*Note*: DFS, disease‐free survival; MSI, microsatellite instability; MSS, microsatellite stability.

For RNA‐seq data of ICGC‐ARGO, HISAT^49^ was used for alignment and RSEM^50^ was used for quantification. The RNA‐seq raw read count from the TCGA database was converted to transcripts per kilobase million (TPM) and further log‐2 transformed. Microarray data were downloaded and preprocessed by the “GEOquery” package.^51^ After removing batch effects through the “ComBat” algorithm, the TCGA‐CRC cohort was merged from the TCGA‐COAD and TCGA‐READ datasets, and the Meta‐GEO cohort was combined from GSE17538, GSE14333, GSE33113, and GSE37892.

### 4.2 Identification of the core senescent genes

The differentially expressed genes (DEGs) between CRC and normal colorectal tissues were screened via the “DESeq2” package. The significant criteria for determining DEGs were set as the *P* < 0.05 and the |log fold change| > 1. We also performed a univariate Cox analysis with all genes in CRC from the ICGC‐ARGO dataset and obtained survival genes associated with DFS. Besides, we obtained 1,499 senescent genes from Peters, et.al.’ s research.^26^ To identify the core senescent genes (CSGs) in CRC, we intersected the DEGs, survival genes and senescent genes, and finally established the CSGs of CRC.

### 4.3 Unsupervised clustering for the senescence subtypes

Unsupervised clustering analysis was applied to identify distinct senescence subtypes based on the expression of CSGs. This process was performed in R using the “ConsensusClusterPlus” package (CCP)^52^ and the “Nbclust” algorithm^53^ was used to determine the optimal number of clusters. Uniform Manifold Approximation and Projection (UMAP), as a common dimension reduction method, was conducted to visualize the differences between these groups in the feature space. To predict the senescence subtypes in other cohorts, we used the DeepCC algorithm.^27^

### 4.4 Estimation of the TME cell infiltration

Single‐sample gene set enrichment analysis (ssGSEA) was employed to quantify the relative abundance of each cell infiltration. Analysis of the gene set of each TME infiltrating immune cell type was obtained from the study of Charoentong et al.^54^ ESTIMATE algorithm was further performed to verify the results of ssGSEA. Besides, to reveal the tumor‐immune interaction and predict the benefit of immunotherapy, we analyzed the relevant molecules or pathways such as immune checkpoint genes, immune suppression signatures, immune exclusion signatures and immune exhaustion signatures by the “IOBR” R package.^33^

### 4.5 Mutation landscape of CRC senescence subtypes

The somatic mutation data were acquired by the “TCGAbiolinks^55^” R package. The mutation landscape was depicted using the functions of the “maftools^56^” R package. We focused on the proportion of genes with mutations in different senescence subtypes, and we compared the differences in the top 10 mutated genes in the three senescence subtypes using the chi‐square test. Since existing studies have reported that exclusive or co‐occurring somatic changes in cancer can indicate functional interactions,^57^, ^58^ we also explored the mutual exclusion or coexistence of genes in three senescence subtypes. According to a previous study,^32^ 10 canonical signaling pathways consisting of 335 genes were evaluated as oncogenic pathways (Table S4). For each patient, we employed ssGSEA algorithms to quantify the enriched abundance of the ten canonical signaling pathways.^59^

### 4.6 Drug sensitivity analysis for senescence subtypes

To further explore the clinical application of the CRC senescence subtypes and our model, we used GDSC^60^ to analyze the differences in drugs among them. These drugs included chemotherapy drugs, anti‐aging drugs, and targeted small‐molecule inhibitors. Moreover, to assess the utility of senescence subtypes in predicting PD‐L1 inhibitor's benefits, we enrolled the IMvigor210 dataset treated with atezolizumab,^61^ of which included 230 non‐responders and 68 responders.

### 4.7 Construction of the senescence scoring system

To better quantify and translate cellular senescence into clinical practice, we developed a senescence scoring system using the least absolute shrinkage and selection operator (LASSO) algorithm. A total of 77 CSGs were included to narrow down and further develop the senescence scoring system. The penalty parameter λ value of the LASSO model was selected based on tenfold cross‐validation, and lambda.1se was used to avoid overfitting. By weighted summation of the expression value of feature genes and their corresponding coefficients obtained from the model, the senescence score was generated. The optimal cut‐off value calculated by the “survminer” package^62^ was used to divide patients into two risk groups with high senescence scores and low senescence scores, respectively.

### 4.8 H&E image analysis and tissue classification

In this study, we retrieved 581 H&E slides of 581 CRC patients from the TCGA cohort (COAD and READ) and use a pathological tile tissue classification algorithm to explore their composition ratio in different senescence subtypes. The classification model was trained and validated on the two public datasets, NCT‐CRC‐HE‐100k and CRC‐VAL‐HE‐7K from https://doi.org/10.5281/zenodo.1214456.^63^ Nine tissue classes were predicted by the classifier, including adipose tissue, background, debris, LYM, mucus, smooth muscle, normal colorectal mucosa, STR and TUM. For inference on the TCGA cohort, all H&E stained WSIs were tessellated into non‐overlapping 224 × 224 pixels image tiles at 20x magnification with Openslides (https://openslide.org/). Each tile was normalized with the Macenko method^64^ and its tissue class was predicted according to the classifier.

### 4.9 Immunohistochemistry

Tissue sections from CRC specimens were deparaffinized in xylene, rehydrated through graded ethanol, and subjected to antigen retrieval by heating in sodium citrate (pH 6.0) in a microwave oven for 15 min. Endogenous peroxidase activity was blocked by incubation in 3% hydrogen peroxide. After blocking with 20% goat serum for 30 min at room temperature, the sections were incubated overnight at 4°C with the primary antibodies (Table S5). The EnVision Plus System‐HRP (DAB; DAKO) was used according to the manufacturer's instructions, and the sections were counterstained with Mayer's hematoxylin.^65^ The stained tumor sections were evaluated by two pathologists, scoring the percentage of positively stained tumor cells and staining intensity as described previously.^66^

### 4.10 Statistical analysis

For the comparison of continuous variables between two groups, the statistical significance of variables was analyzed using the Wilcoxon rank sum test, while the Kruskal Wallis test was used to assess the significant difference for more than two group comparisons. The chi‐square test was used to compare categorical variables. Cox regression and Kaplan‐Meier analysis were conducted by the “survival” R package. The “survminer” R package was performed to determine the optimal cut‐off value. A *p‐*value of less than 0.05 was considered statistically significant. All processing was done in the R 4.1.1 software.

## AUTHOR CONTRIBUTIONS

M.Y.L., D.C., C.H.L., and J.G.C. contributed equally to this study. M.Y.L., D.C., C.H.L, J.G.C., P.L., X.J.W., X.S.H., and F.G. contributed to the study concept and design, the acquisition, analysis, and interpretation of data, and the drafting of the manuscript. G.M.L., C.L.H., B.W.G., and J.X.L. contributed to the data collection and manuscript reviews. All authors have read and approved the final manuscript.

## CONFLICT OF INTEREST STATEMENT

The authors declare no conflict of interest.

## ETHICS STATEMENT

The research scheme was approved by the Ethics Review Committee of the Sixth Affiliated Hospital, Sun Yat‐sen University (2022ZSLYEC‐362).

## Supporting information



Supporting Information

## Data Availability

The data generated in this study are publicly available in Gene Expression Omnibus (GEO) at GSE17538, GSE14333, GSE33113, and GSE37892. ICGC‐ARGO RNA‐seq data are not publicly available but are available upon reasonable request from the corresponding author.
