# Supplementary material for: Senescence‐based colorectal cancer subtyping reveals distinct molecular characteristics and therapeutic strategies
Source: MedComm (2020). 2023 Jul 26;4(4):e333. doi: 10.1002/mco2.333 (PMC10369159; doi:10.1002/mco2.333)
Supplement: Supplementary file 1 — Supporting Information [file MCO2-4-e333-s001.pdf]

Senescence-based Colorectal Cancer Subtyping Reveals Distinct Molecular Characteristics and Therapeutic Strategies

Min-Yi Lv[1,2,3#], Du Cai[1,2,3#], Cheng-Hang Li[1,2,3#], Junguo Chen[1,2,3#], Guanman Li[1,2,3], Chuling Hu[1,2,3], Baowen Gai[1,2,3], Jiaxin Lei[1,2,3], Ping Lan[1,2,3*], Xiaojian Wu[1,2,3*], Xiaosheng He[1,2,3*], Feng Gao[1,2,3*]

[1]Department of Colorectal Surgery, The Sixth Affiliated Hospital, Sun Yat-sen University, Guangzhou, China

[2] Guangdong Institute of Gastroenterology, Guangzhou, China

[3] Guangdong Provincial Key Laboratory of Colorectal and Pelvic Floor Disease, The Sixth Affiliated Hospital, Sun Yat-sen University, Guangzhou, China

# These authors contributed equally to this article.

Corresponding Authors: Ping Lan, lanping@mail.sysu.edu.cn; Xiaojian Wu, wuxjian@mail.sysu.edu.cn; Xiaosheng He, hexsheng@mail.sysu.edu.cn; Feng Gao, gaof57@mail.sysu.edu.cn.

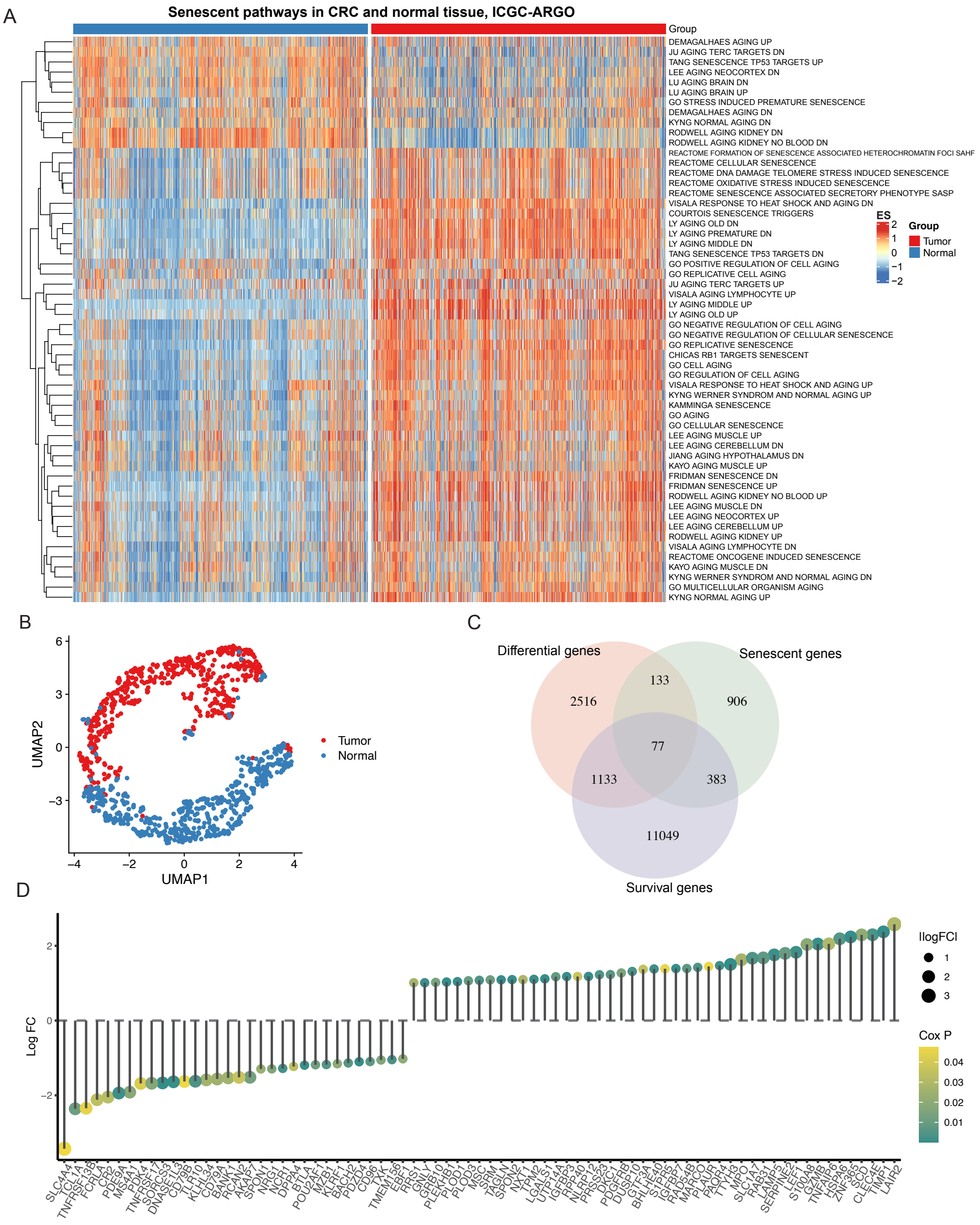

Supplementary Fig.S1 Identification of the senescence feature in colorectal cancer (CRC). (A) Heatmap showed significant enrichment of senescence-related pathways in CRC compared to normal tissue in the ICGC-ARGO cohort. ES: enrichment scores. **(B)** The UMAP plot showed that senescence-related pathways could distinguish CRC from normal tissue in the ICGC-ARGO cohort. **(C)** Venn diagram showed that the core senescent genes (CSGs) were established by overlapping among the DEGs, senescence genes and survival genes. **(D)** The dot plot showed the correlation between CSGs and their *P*-values in relation to disease-free survival (DFS).

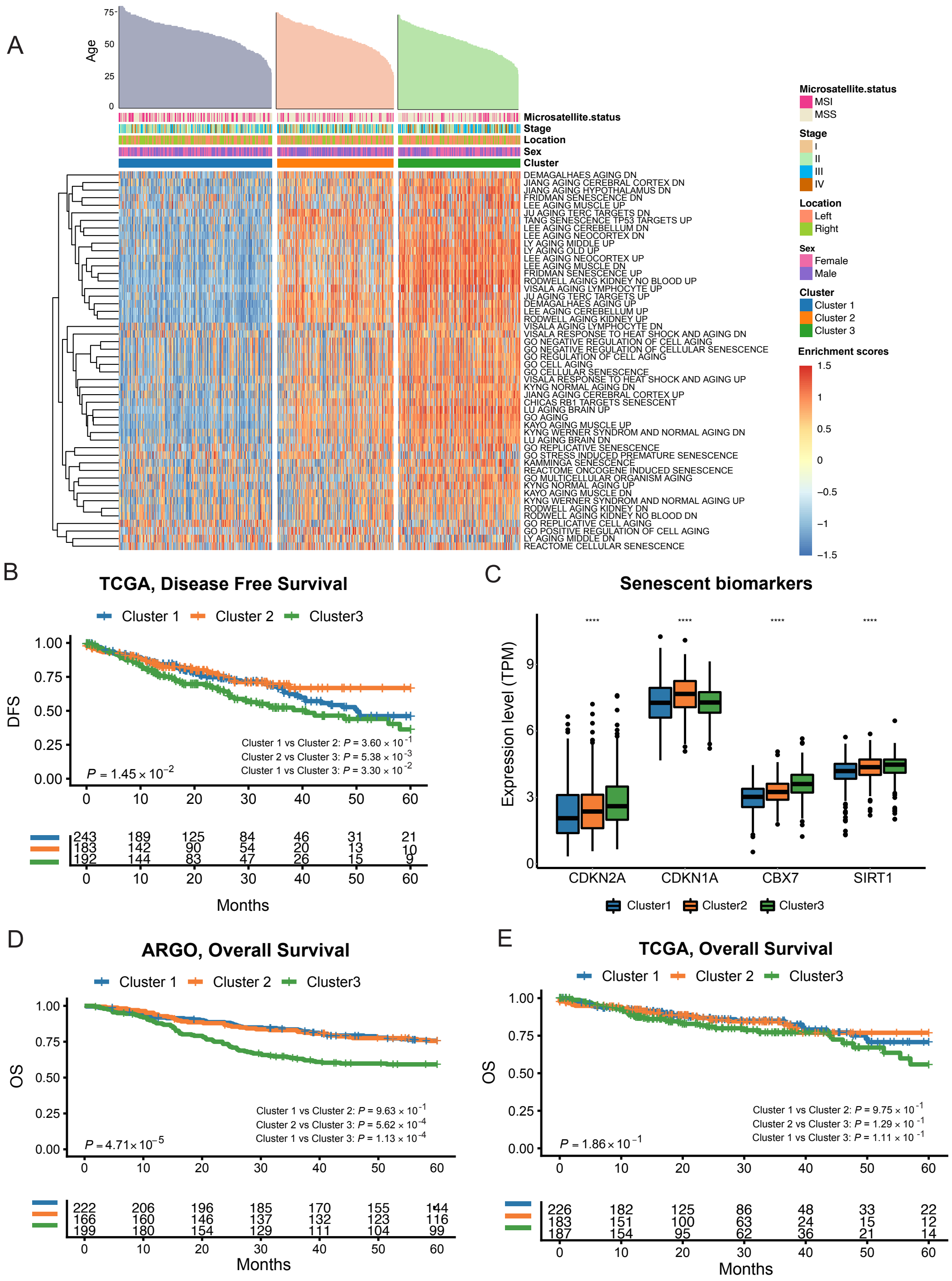

Supplementary Fig.S2 Validation of the senescence subtypes in the TCGA-CRC cohort. (A) Heatmap showed the enrichment of senescence-related pathways of three senescence subtypes in the TCGA-CRC cohort. Age, sex, tumor location, TNM stage and microsatellite status were shown above the heatmap. The asterisks represented the statistical *P* value (ns, not significant; *$P < 0.05$; **$P < 0.01$; ***$P < 0.001$; **** $P < 0.0001$). **(B)** Survival analyses for the three senescence subtypes in the TCGA-CRC cohort. Kaplan–Meier curves showed a significant disease-free survival

difference among three senescence subtypes. **(C)** Difference of senescent biomarkers among three senescence subtypes in the TCGA-CRC cohort. **(D)** Survival analyses for the three senescence subtypes in the ICGC-ARGO cohort. Kaplan–Meier curves showed a significant overall survival difference among three senescence subtypes. **(E)** Survival analyses for the three senescence subtypes in the TCGA-CRC cohort. Kaplan–Meier curves showed a significant overall survival difference among three senescence subtypes.

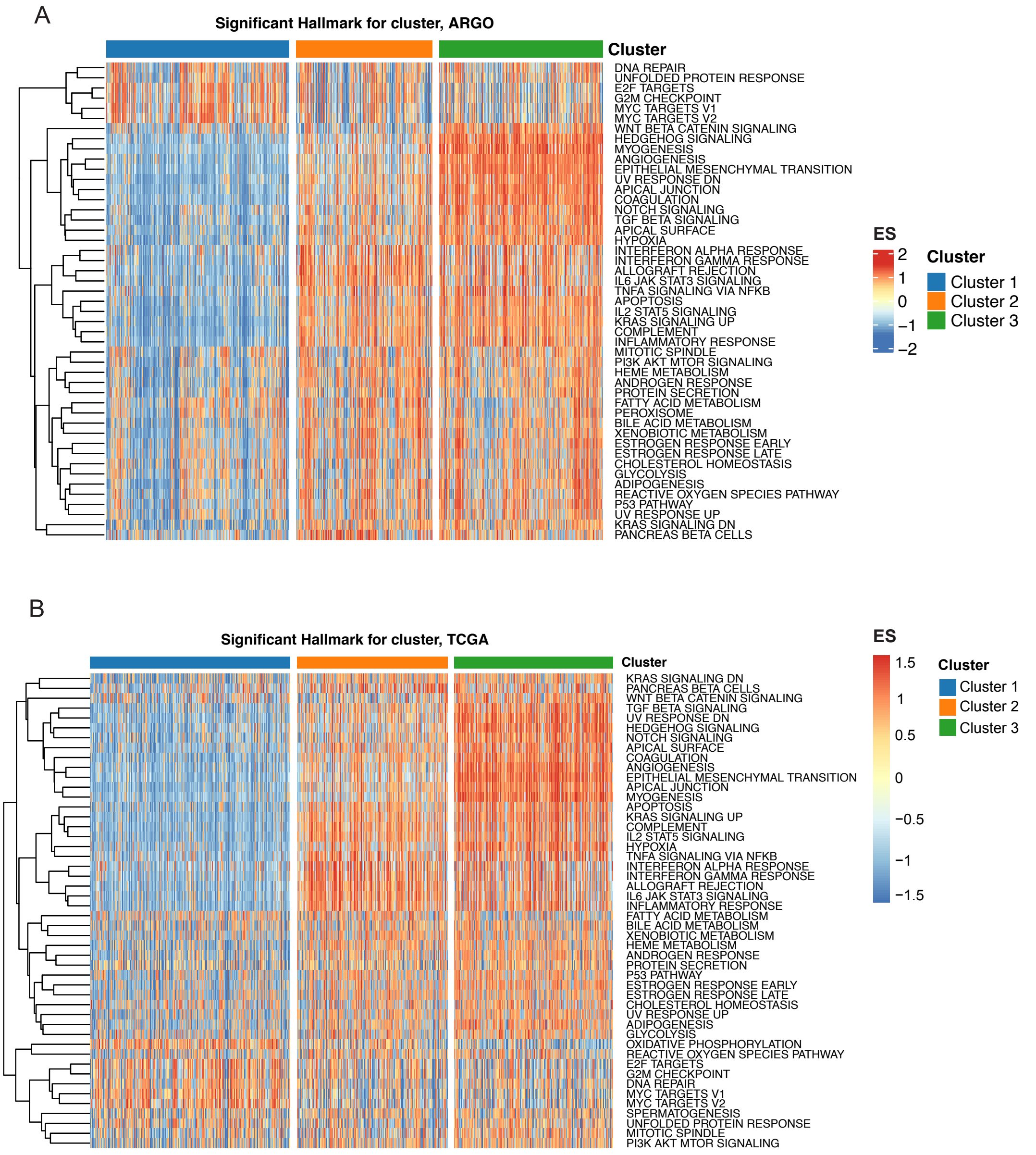

Supplementary Fig.S3 The Hallmark of colorectal cancer. (A, B) Heatmap showed the enrichment of hallmarks of three senescence subtypes in the ICGC-ARGO cohort **(A)** and TCGA-CRC cohort **(B)**. ES: enrichment scores.

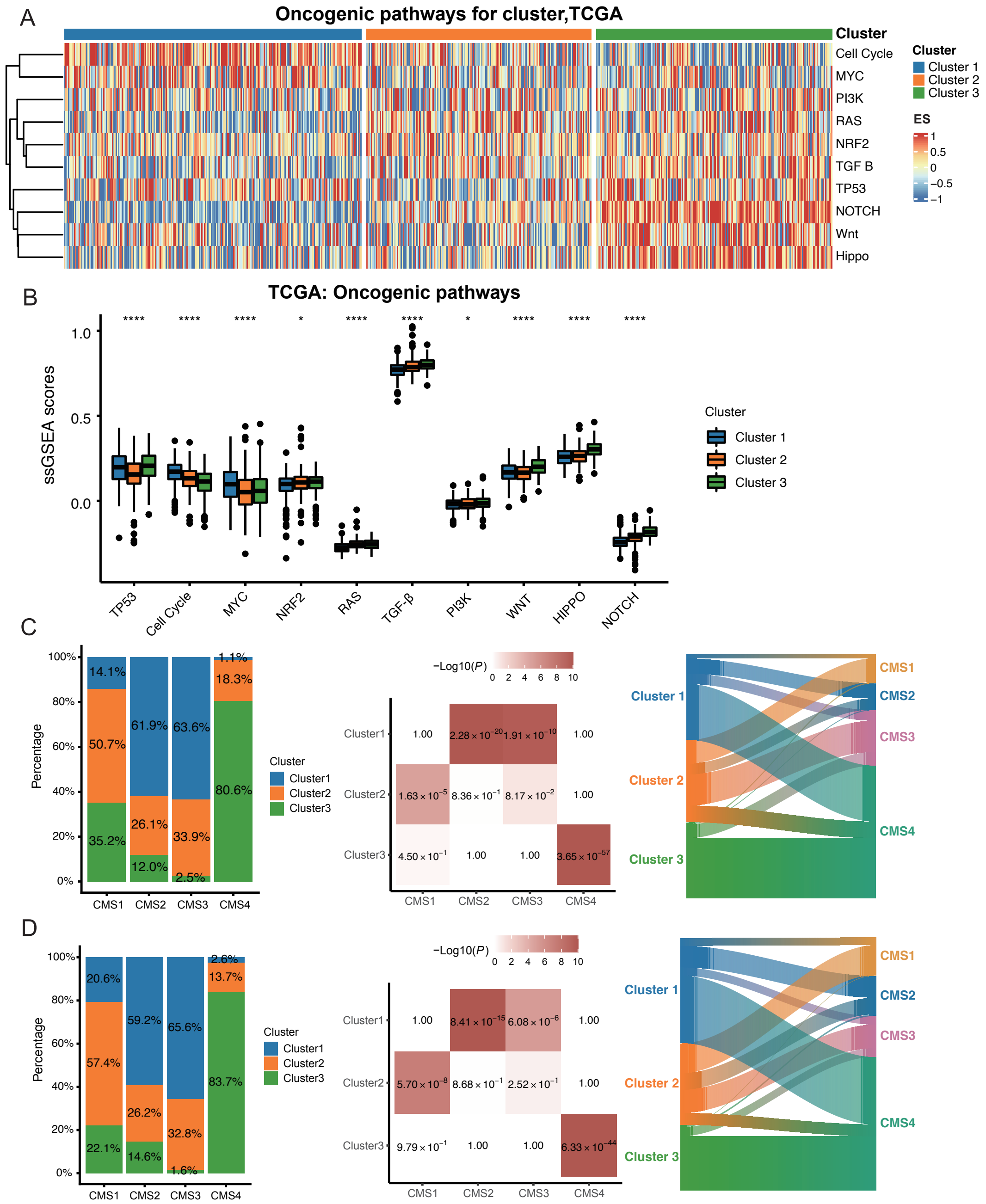

Supplementary Fig.S4 Validation of oncogenic pathways in three senescence subtypes. (A) Heatmap showed the enrichment of ten important oncogenic pathways among the three senescence subtypes in the TCGA-CRC cohort. ES: enrichment scores. **(B)** Boxplot showed score variations in ten important oncogenic pathways among the three senescence subtypes in TCGA- CRC cohort. **(C, D)** Sankey plot showed the correlation between senescence subtypes and consensus molecular subtypes (CMS) in the ICGC-ARGO cohort **(C)** and TCGA-CRC cohort **(D)**.

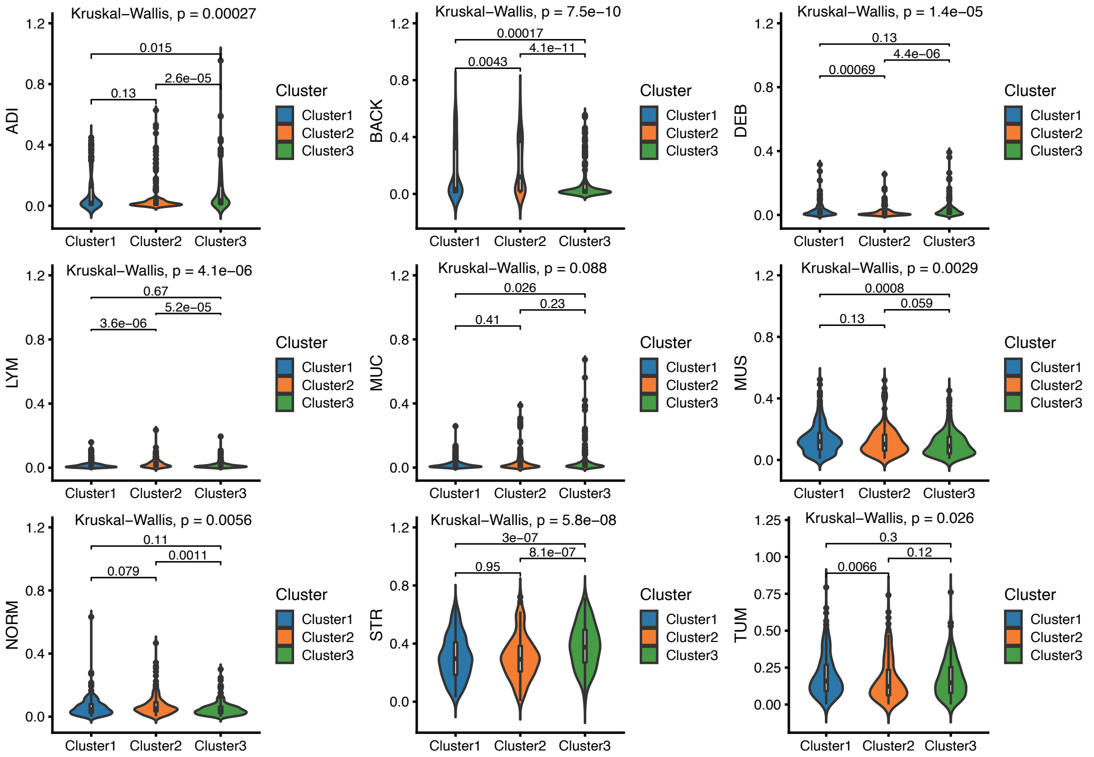

Supplementary Fig.S5 Nine tissues distribution of three Clusters. The violin plot showed the results of the nine tissue classes algorithm in predicting the number of cells in the three senescence subtypes (TCGA Pathology Slide).

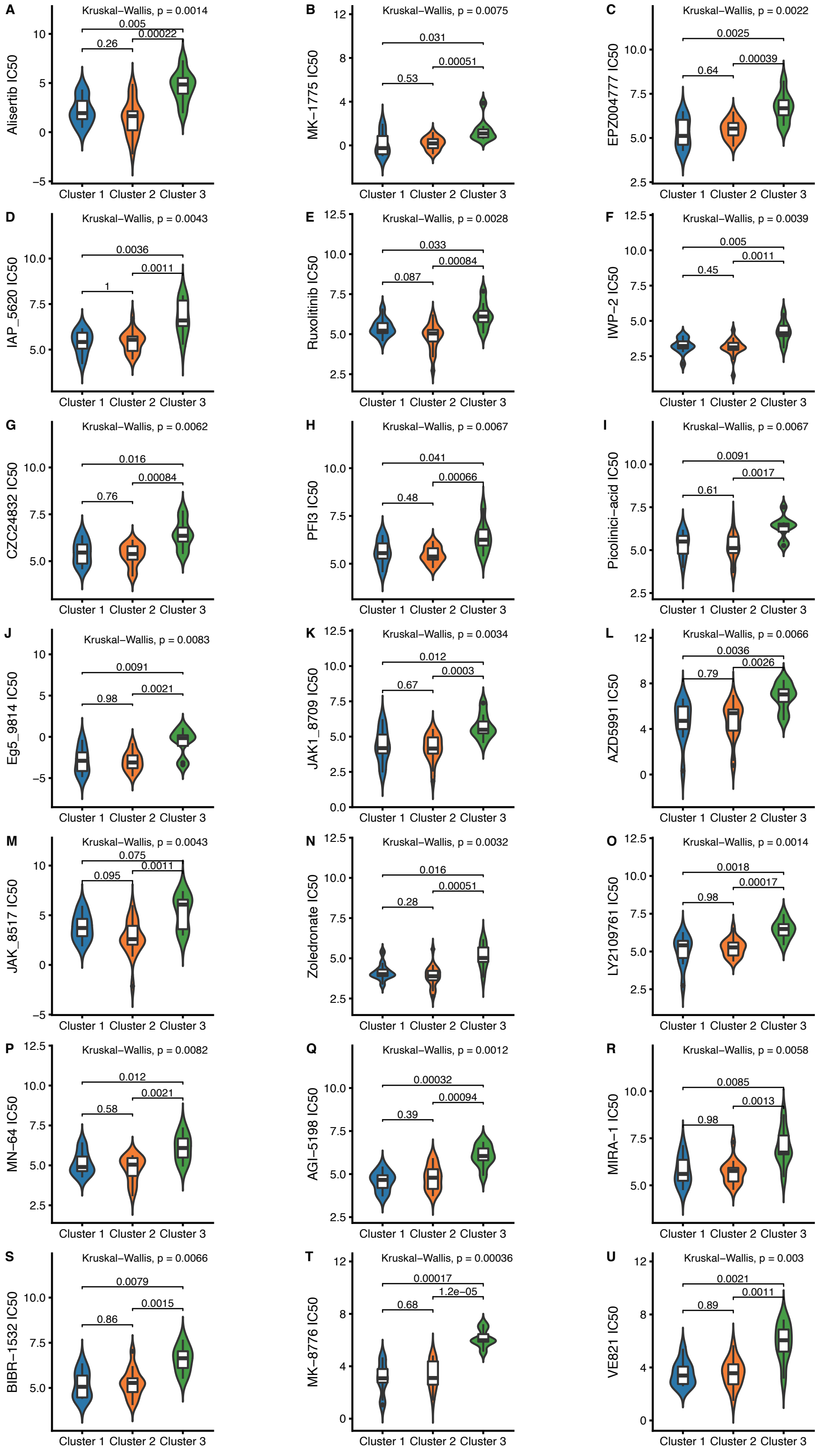

Supplementary Fig.S6 Statistically significant drugs in three senescence subtypes. The violin plot showed all the drugs in the GDSC database with statistical significance among the three senescence subtypes.

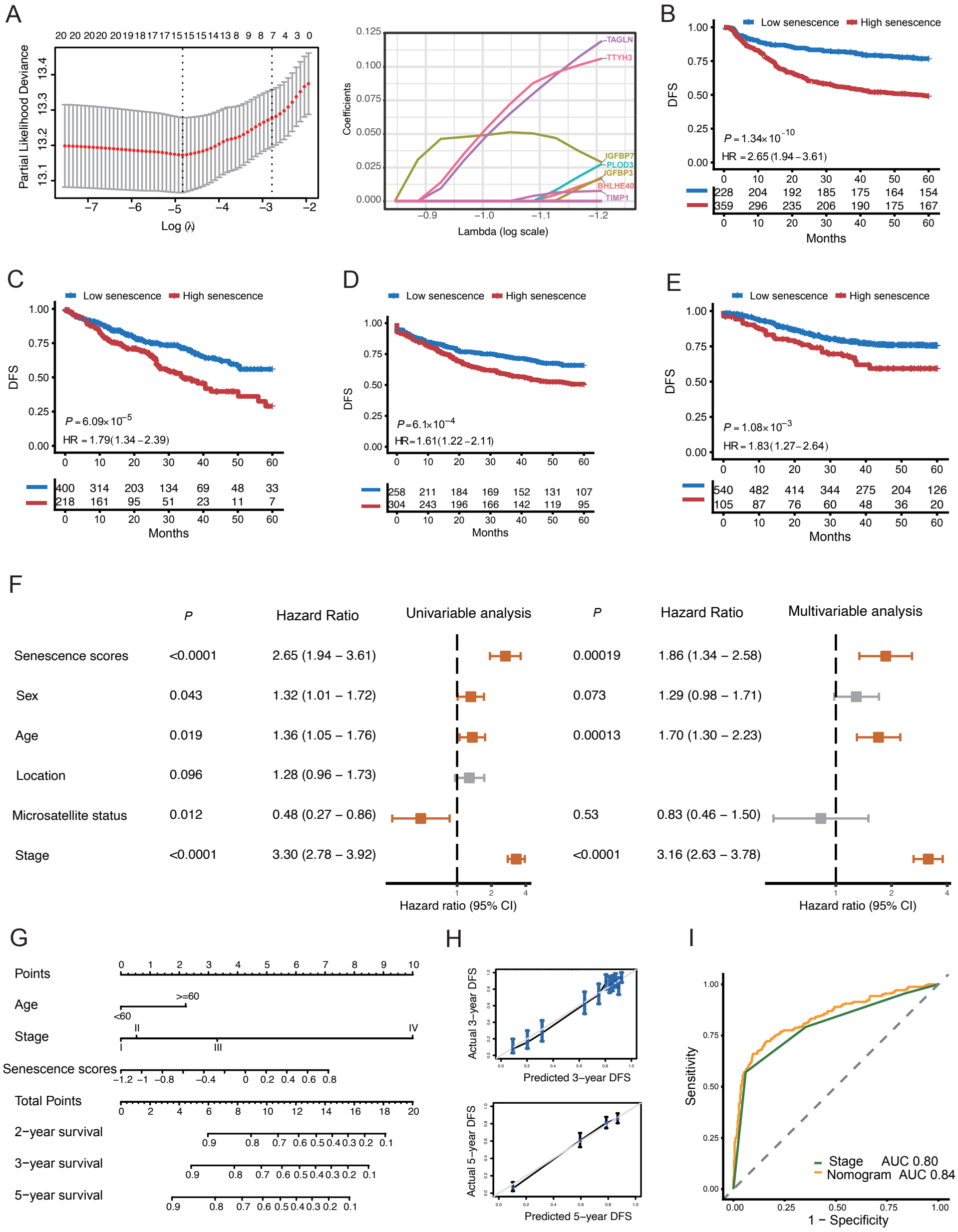

Supplementary Fig.S7 Construction of the senescence score with clinical utility. (A) Identification of the 7 CSGs by LASSO algorithm and construction of the senescence score model. **(B-E)** Kaplan–Meier curves comparing disease-free survival (DFS) of patients with the low and high senescence scores in the ICGC-ARGO **(B)**, TCGA-CRC **(C)**, GSE39582 **(D)** and Meta-GEO **(E)** cohorts. **(F)** The Forest plot showed the univariate and multivariate Cox regression analyses of senescence scores and other clinical features in the ICGC-ARGO cohort. **(G)** Nomogram was developed in the ICGC-ARGO cohort based on the senescence score, age and TNM stage to predict 2-year, 3-year, and 5-year outcomes. **(H)** Calibration curves of 3-year and 5-year DFS for patients with CRC in the ICGC-ARGO cohort. **(I)** Time-dependent ROC of DFS for patients with CRC in the ICGC-ARGO cohort.

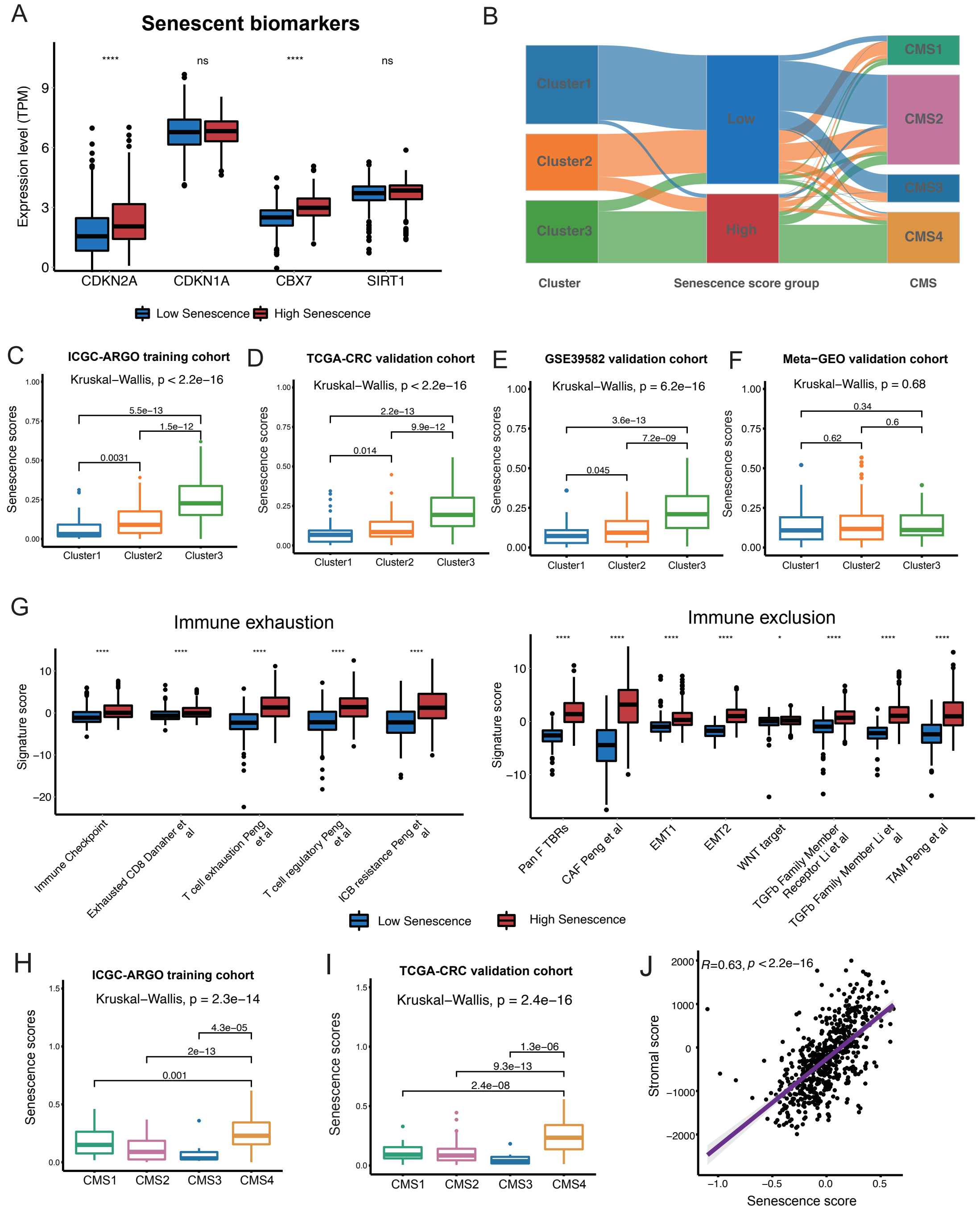

Supplementary Fig.S8 Validation of the senescence score group in the TCGA-CRC cohort. (A) Difference of senescent biomarkers between high and low senescence score groups (TCGA-CRC cohort). **(B)** Sankey plot showed the correlation between senescent subtypes, senescence score groups and consensus molecular subtypes (CMS) (TCGA-CRC cohort). **(C-F)** The correlation between senescence subtypes and senescence scores in the ICGC-ARGO cohort **(C)**, TCGA-CRC cohort **(D)**, GSE39582 **(E)** and Meta-GEO cohort **(F)**. **(G)** Boxplot showed the differences in

immune exclusion and immune exhaustion between high and low senescence score groups in the TCGA-CRC cohort. **(H, I)** The correlation between CMS and senescence scores in the ICGC-ARGO cohort **(H)** and TCGA-CRC cohort **(I)**. **(J)** The correlation between stromal score and senescence score in the ICGC-ARGO cohort.

Supplementary Table 1. The summary of senescence pathways

**Senescence Pathways**
CHICAS_RB1_TARGETS_SENESCENT
COURTOIS_SENESCENCE_TRIGGERS
DEMAGALHAES_AGING_DN
DEMAGALHAES_AGING_UP
FRIDMAN_SENESCENCE_DN
FRIDMAN_SENESCENCE_UP
GO_AGING
GO_CELL_AGING
GO_CELLULAR_SENESCENCE
GO_DNA_PACKAGING
GO_DNA_PACKAGING_COMPLEX
GO_MULTICELLULAR_ORGANISM_AGING
GO_NEGATIVE_REGULATION_OF_CELL_AGING
GO_NEGATIVE_REGULATION_OF_CELLULAR_SENESCENCE
GO_POSITIVE_REGULATION_OF_CELL_AGING
GO_REGULATION_OF_CELL_AGING
GO_REPLICATIVE_CELL_AGING
GO_REPLICATIVE_SENESCENCE
GO_STRESS_INDUCED_PREMATURE_SENESCENCE
JIANG_AGING_CEREBRAL_CORTEX_DN
JIANG_AGING_CEREBRAL_CORTEX_UP
JIANG_AGING_HYPOTHALAMUS_DN
JIANG_AGING_HYPOTHALAMUS_UP
JU_AGING_TERC_TARGETS_DN
JU_AGING_TERC_TARGETS_UP
KAMMINGA_SENESCENCE
KAYO_AGING_MUSCLE_DN
KAYO_AGING_MUSCLE_UP
KYNG_NORMAL_AGING_DN
KYNG_NORMAL_AGING_UP

KYNG_WERNER_SYNDROM_AND_NORMAL_AGING_DN
KYNG_WERNER_SYNDROM_AND_NORMAL_AGING_UP
LEE_AGING_CEREBELLUM_DN
LEE_AGING_CEREBELLUM_UP
LEE_AGING_MUSCLE_DN
LEE_AGING_MUSCLE_UP
LEE_AGING_NEOCORTEX_DN
LEE_AGING_NEOCORTEX_UP
LU_AGING_BRAIN_DN
LU_AGING_BRAIN_UP
LY_AGING_MIDDLE_DN
LY_AGING_MIDDLE_UP
LY_AGING_OLD_DN
LY_AGING_OLD_UP
LY_AGING_PREMATURE_DN
REACTOME_CELLULAR_SENESCENCE
REACTOME_DNA_DAMAGE_TELOMERE_STRESS_INDUCED_SENESCENCE
REACTOME_FORMATION_OF_SENESCENCE_ASSOCIATED_HETEROCHROMATIN_FOCI_SAHF
REACTOME_NICOTINAMIDE_SALVAGING
REACTOME_ONCOGENE_INDUCED_SENESCENCE
REACTOME_OXIDATIVE_STRESS_INDUCED_SENESCENCE
REACTOME_SENESCENCE_ASSOCIATED_SECRETORY_PHENOTYPE_SASP
RODWELL_AGING_KIDNEY_DN
RODWELL_AGING_KIDNEY_NO_BLOOD_DN
RODWELL_AGING_KIDNEY_NO_BLOOD_UP
RODWELL_AGING_KIDNEY_UP
TANG_SENESCENCE_TP53_TARGETS_DN
TANG_SENESCENCE_TP53_TARGETS_UP
VISALA_AGING_LYMPHOCYTE_DN
VISALA_AGING_LYMPHOCYTE_UP
VISALA_RESPONSE_TO_HEAT_SHOCK_AND_AGING_DN
VISALA_RESPONSE_TO_HEAT_SHOCK_AND_AGING_UP

Supplementary Table 2. Details of statistically significant drugs in the three senescence subtypes.

Drug	Synonyms	Target	Target pathways	Sensetive senescence subtypes
Ruxolitinib	INCB-18424, Ruxolitinib Phosphate, Jakafi	JAK1, JAK2	Other, kinases	Cluster1,Cluster2
Sepantronium bromide	YM155, YM-155, YM 155	BIRC5	Apoptosis regulation	Cluster3

Alisertib	MLN8237	AURKA	Mitosis	Cluster1,Cluster2
Docetaxel	RP-56976, Taxotere	Microtubule stabiliser	Mitosis	Cluster1,Cluster2
MK-1775	AZD1775	WEE1, PLK1	Cell cycle	Cluster1,Cluster2
EPZ004777	EPZ-004777	DOT1L	Chromatin histone methylation	Cluster1,Cluster2
IAP_5620	SN1043546339	IAP	Other	Cluster1,Cluster2
IWP-2	Wnt Inhibitor IWP-2	PORCN	WNT signaling	Cluster1,Cluster2
VE-822	VE 822, VE822, Berzosertib	ATR	Genome integrity	Cluster1
CZC24832	GTPL6653	PI3Kgamma	PI3K/MTOR signaling	Cluster1,Cluster2
PFI3	PFI-3, PFI 3, AOB2221	Polybromo 1, SMARCA4, SMARCA2	Chromatin other	Cluster1,Cluster2
Picolinici-acid	Picolinate	Inflammatory related	Other	Cluster1,Cluster2
Eg5_9814	SN1047613775, Eg5_9814	KSP11	Other	Cluster1,Cluster2
JAK1_8709	SN1049200060, JAK1_8709	JAK1	Other, kinases	Cluster1,Cluster2
AZD5991	SN1049446612, AZD5991	MCL1	Apoptosis regulation	Cluster1,Cluster2
JAK_8517	SN1066590414, JAK_8517	JAK1, JAK2	Other, kinases	Cluster1,Cluster2
Zoledronate	Zoledronic acid, Zometa, Reclast		Unclassified	Cluster1,Cluster2
Carmustine			DNA replication	Cluster1,Cluster2
LY2109761	5XE	TGFB1	Other	Cluster1,Cluster2
MN-64		TNKS1, TNKS2	WNT signaling	Cluster1,Cluster2
AGI-5198	IDH-C35	IDH1 (R132H)	Metabolism	Cluster1,Cluster2
MIRA-1	MIRA 1, MIRA1, NSC19630, NSC-19630, NSC 19630	TP53	p53 pathway	Cluster1,Cluster2
BIBR-1532		TERT	Genome integrity	Cluster1,Cluster2
MK-8776	SCH900776	CHEK1, CHEK2, CDK2	Cell cycle	Cluster1,Cluster2

VE821	VE 821, VE-821	ATR	Genome integrity	Cluster1,Cluster2

Supplementary Table 3. Univariate Cox analysis of clinical factors related to DFS of patients in ICGC-ARGO cohort

Variable	HR	95CI%（low）	95CI%（high）	adjusted *P*
Senescence scores	2.65	1.94	3.61	<0.0001
Sex	1.32	1.01	1.72	0.043
Age	1.36	1.05	1.76	0.019
Tumor location	1.28	0.96	1.73	0.096
Microsatellite status	0.48	0.27	0.86	0.012
TNM stage	3.3	2.78	3.92	<0.0001

Supplementary Table 3. Multivariate Cox analysis of clinical factors related to DFS of patients in ICGC-ARGO cohort

Variable	HR	95CI%（low）	95CI%（high）	adjusted *P*
Senescence scores	1.86	1.34	2.58	0.00019
Sex	1.29	0.98	1.71	0.073
Age	1.7	1.3	2.23	0.00013
Tumor location	NA	NA	NA	NA
Microsatellite status	0.83	0.46	1.5	0.53
TNM stage	3.16	2.63	3.78	<0.0001

Supplementary Table 4. The signatures of ten oncogenic pathways

**Symbol**	**Pathway**
CCND1	Cell.Cycle_activated
CCND2	Cell.Cycle_activated
CCND3	Cell.Cycle_activated
CCNE1	Cell.Cycle_activated
CDK2	Cell.Cycle_activated
CDK4	Cell.Cycle_activated
CDK6	Cell.Cycle_activated
E2F1	Cell.Cycle_activated
E2F3	Cell.Cycle_activated
YAP1	Hippo_activated
TEAD1	Hippo_activated

TEAD2	Hippo_activated
TEAD3	Hippo_activated
TEAD4	Hippo_activated
WWTR1	Hippo_activated
MYC	MYC_activated
MYCL1	MYC_activated
MYCN	MYC_activated
CREBBP	NOTCH_activated
EP300	NOTCH_activated
HES1	NOTCH_activated
HES2	NOTCH_activated
HES3	NOTCH_activated
HES4	NOTCH_activated
HES5	NOTCH_activated
HEY1	NOTCH_activated
HEY2	NOTCH_activated
HEYL	NOTCH_activated
KAT2B	NOTCH_activated
NOTCH1	NOTCH_activated
NOTCH2	NOTCH_activated
NOTCH3	NOTCH_activated
NOTCH4	NOTCH_activated
PSEN2	NOTCH_activated
LFNG	NOTCH_activated
NCSTN	NOTCH_activated
JAG1	NOTCH_activated
APH1A	NOTCH_activated
FHL1	NOTCH_activated
THBS2	NOTCH_activated
MFAP2	NOTCH_activated
RFNG	NOTCH_activated
MFAP5	NOTCH_activated
JAG2	NOTCH_activated
MAML3	NOTCH_activated
MFNG	NOTCH_activated
CNTN1	NOTCH_activated
MAML1	NOTCH_activated
MAML2	NOTCH_activated
PSEN1	NOTCH_activated
PSENEN	NOTCH_activated
RBPJ	NOTCH_activated
RBPJL	NOTCH_activated

SNW1	NOTCH_activated
ADAM10	NOTCH_activated
APH1B	NOTCH_activated
ADAM17	NOTCH_activated
DLK1	NOTCH_activated
DLL1	NOTCH_activated
DLL3	NOTCH_activated
DLL4	NOTCH_activated
DNER	NOTCH_activated
DTX1	NOTCH_activated
DTX2	NOTCH_activated
DTX3	NOTCH_activated
DTX3L	NOTCH_activated
DTX4	NOTCH_activated
EGFL7	NOTCH_activated
NFE2L2	NRF2_activated
EIF4EBP1	PI3K_activated
AKT1	PI3K_activated
AKT2	PI3K_activated
AKT3	PI3K_activated
AKT1S1	PI3K_activated
INPP4B	PI3K_activated
MAPKAP1	PI3K_activated
MLST8	PI3K_activated
MTOR	PI3K_activated
PDK1	PI3K_activated
PIK3CA	PI3K_activated
PIK3CB	PI3K_activated
PIK3R2	PI3K_activated
RHEB	PI3K_activated
RICTOR	PI3K_activated
RPTOR	PI3K_activated
RPS6	PI3K_activated
RPS6KB1	PI3K_activated
STK11	PI3K_activated
TGFBR1	TGF-B_activated
TGFBR2	TGF-B_activated
ACVR2A	TGF-B_activated
ACVR1B	TGF-B_activated
SMAD2	TGF-B_activated
SMAD3	TGF-B_activated
SMAD4	TGF-B_activated

TP53	TP53_activated
ATM	TP53_activated
CHEK2	TP53_activated
RPS6KA3	TP53_activated
LEF1	Wnt_activated
LGR4	Wnt_activated
LGR5	Wnt_activated
LZTR1	Wnt_activated
NDP	Wnt_activated
PORCN	Wnt_activated
SFRP1	Wnt_activated
SFRP2	Wnt_activated
SFRP4	Wnt_activated
SFRP5	Wnt_activated
SOST	Wnt_activated
TCF7L1	Wnt_activated
WIF1	Wnt_activated
ZNRF3	Wnt_activated
CTNNB1	Wnt_activated
DVL1	Wnt_activated
DVL2	Wnt_activated
DVL3	Wnt_activated
FRAT1	Wnt_activated
FRAT2	Wnt_activated
DKK1	Wnt_activated
DKK2	Wnt_activated
DKK3	Wnt_activated
DKK4	Wnt_activated
RNF43	Wnt_activated
TCF7	Wnt_activated
TCF7L2	Wnt_activated
ABL1	RAS_activated
EGFR	RAS_activated
ERBB2	RAS_activated
ERBB3	RAS_activated
ERBB4	RAS_activated
PDGFRA	RAS_activated
PDGFRB	RAS_activated
MET	RAS_activated
FGFR1	RAS_activated
FGFR2	RAS_activated
FGFR3	RAS_activated

FGFR4	RAS_activated
FLT3	RAS_activated
ALK	RAS_activated
RET	RAS_activated
ROS1	RAS_activated
KIT	RAS_activated
IGF1R	RAS_activated
NTRK1	RAS_activated
NTRK2	RAS_activated
NTRK3	RAS_activated
SOS1	RAS_activated
GRB2	RAS_activated
PTPN11	RAS_activated
KRAS	RAS_activated
HRAS	RAS_activated
NRAS	RAS_activated
RIT1	RAS_activated
ARAF	RAS_activated
BRAF	RAS_activated
RAF1	RAS_activated
RAC1	RAS_activated
MAP2K1	RAS_activated
MAP2K2	RAS_activated
MAPK1	RAS_activated
INSR	RAS_activated
INSRR	RAS_activated
IRS1	RAS_activated
SOS2	RAS_activated
SHC1	RAS_activated
SHC2	RAS_activated
SHC3	RAS_activated
SHC4	RAS_activated
RASGRP1	RAS_activated
RASGRP2	RAS_activated
RASGRP3	RAS_activated
RASGRP4	RAS_activated
RAPGEF1	RAS_activated
RAPGEF2	RAS_activated
RASGRF1	RAS_activated
RASGRF2	RAS_activated
FNTA	RAS_activated
FNTB	RAS_activated

SPRED1	RAS_activated
SPRED2	RAS_activated
SPRED3	RAS_activated
SHOC2	RAS_activated
KSR1	RAS_activated
KSR2	RAS_activated
JAK2	RAS_activated
IRS2	RAS_activated
CDKN1A	Cell.Cycle_repressed
CDKN1B	Cell.Cycle_repressed
CDKN2A	Cell.Cycle_repressed
CDKN2B	Cell.Cycle_repressed
CDKN2C	Cell.Cycle_repressed
RB1	Cell.Cycle_repressed
STK4	Hippo_repressed
STK3	Hippo_repressed
SAV1	Hippo_repressed
LATS1	Hippo_repressed
LATS2	Hippo_repressed
MOB1A	Hippo_repressed
MOB1B	Hippo_repressed
PTPN14	Hippo_repressed
NF2	Hippo_repressed
WWC1	Hippo_repressed
TAOK1	Hippo_repressed
TAOK2	Hippo_repressed
TAOK3	Hippo_repressed
CRB1	Hippo_repressed
CRB2	Hippo_repressed
CRB3	Hippo_repressed
LLGL1	Hippo_repressed
LLGL2	Hippo_repressed
HMCN1	Hippo_repressed
SCRIB	Hippo_repressed
HIPK2	Hippo_repressed
FAT1	Hippo_repressed
FAT2	Hippo_repressed
FAT3	Hippo_repressed
FAT4	Hippo_repressed
DCHS1	Hippo_repressed
DCHS2	Hippo_repressed
CSNK1E	Hippo_repressed

CSNK1D	Hippo_repressed
AJUBA	Hippo_repressed
LIMD1	Hippo_repressed
WTIP	Hippo_repressed
MGA	MYC_repressed
MNT	MYC_repressed
MXD1	MYC_repressed
MXD3	MYC_repressed
MXD4	MYC_repressed
MXI1	MYC_repressed
ARRDC1	NOTCH_repressed
CNTN6	NOTCH_repressed
KDM5A	NOTCH_repressed
NOV	NOTCH_repressed
NRARP	NOTCH_repressed
ITCH	NOTCH_repressed
SPEN	NOTCH_repressed
FBXW7	NOTCH_repressed
HDAC2	NOTCH_repressed
CUL1	NOTCH_repressed
NCOR1	NOTCH_repressed
NCOR2	NOTCH_repressed
HDAC1	NOTCH_repressed
NUMB	NOTCH_repressed
CIR1	NOTCH_repressed
NUMBL	NOTCH_repressed
RBX1	NOTCH_repressed
SAP30	NOTCH_repressed
SKP1	NOTCH_repressed
CTBP1	NOTCH_repressed
CTBP2	NOTCH_repressed
KEAP1	NRF2_repressed
CUL3	NRF2_repressed
DEPDC5	PI3K_repressed
DEPTOR	PI3K_repressed
NPRL2	PI3K_repressed
NPRL3	PI3K_repressed
PIK3R1	PI3K_repressed
PIK3R3	PI3K_repressed
PPP2R1A	PI3K_repressed
PTEN	PI3K_repressed
TSC1	PI3K_repressed

TSC2	PI3K_repressed
NF1	RAS_repressed
RASA1	RAS_repressed
CBL	RAS_repressed
ERRFI1	RAS_repressed
CBLB	RAS_repressed
CBLC	RAS_repressed
RCE1	RAS_repressed
ICMT	RAS_repressed
MRAS	RAS_repressed
PLXNB1	RAS_repressed
MAPK3	RAS_repressed
ARHGAP35	RAS_repressed
RASA2	RAS_repressed
RASA3	RAS_repressed
RASAL1	RAS_repressed
RASAL2	RAS_repressed
RASAL3	RAS_repressed
DAB2IP	RAS_repressed
PPP1CA	RAS_repressed
SCRIB	RAS_repressed
PIN1	RAS_repressed
PEBP1	RAS_repressed
ERF	RAS_repressed
PEA15	RAS_repressed
MDM2	TP53_repressed
MDM4	TP53_repressed
CHD8	Wnt_repressed
LRP5	Wnt_repressed
LRP6	Wnt_repressed
RSPO1	Wnt_repressed
TLE1	Wnt_repressed
TLE2	Wnt_repressed
TLE3	Wnt_repressed
TLE4	Wnt_repressed
FZD1	Wnt_repressed
FZD10	Wnt_repressed
FZD2	Wnt_repressed
FZD3	Wnt_repressed
FZD4	Wnt_repressed
FZD5	Wnt_repressed
FZD6	Wnt_repressed

FZD7	Wnt_repressed
FZD8	Wnt_repressed
FZD9	Wnt_repressed
WNT1	Wnt_repressed
WNT10A	Wnt_repressed
WNT10B	Wnt_repressed
WNT11	Wnt_repressed
WNT16	Wnt_repressed
WNT2	Wnt_repressed
WNT3A	Wnt_repressed
WNT4	Wnt_repressed
WNT5A	Wnt_repressed
WNT5B	Wnt_repressed
WNT6	Wnt_repressed
WNT7A	Wnt_repressed
WNT7B	Wnt_repressed
WNT8A	Wnt_repressed
WNT8B	Wnt_repressed
WNT9A	Wnt_repressed
WNT9B	Wnt_repressed
AMER1	Wnt_repressed
APC	Wnt_repressed
AXIN1	Wnt_repressed
AXIN2	Wnt_repressed
GSK3B	Wnt_repressed
CHD4	Wnt_repressed

Supplementary Table 5. Information of utilized antibodies.

Antibody	Catalogue Numbers	Dilution	Manufacturer
CDKN2A	10883-1-AP	1:1000	Proteintech (Wuhan, China)
CDKN1A	10355-1-AP	1:200	Proteintech (Wuhan, China)
SIRT1	8469S	1:200	Cell Signaling Technology (Danvers, MA, US)
CBX7	ab21873	1:1000	Abcam (Cambridge, MA, US)
